# A new type of bladeless turbine for compressed gas energy storage system

**DOI:** 10.3389/fchem.2022.1013473

**Published:** 2022-09-26

**Authors:** Qin Wang, Zhengyang Zhu, Wei Chen, Yang Zhou

**Affiliations:** ^1^ Key Laboratory of Energy Thermal Conversion and Control of Ministry of Education, School of Energy and Environment, Southeast University, Nanjing, China; ^2^ School of Mechanical Engineering, Wanjiang University of Technology, Maanshan, China; ^3^ Jiangsu Jiaoke Energy Technology Development Co., Ltd., Nanjing, China

**Keywords:** vaneless turbine, rotor dynamics, fluent, bench test, energy storage

## Abstract

Nowadays, energy storage engineering is an important means to relieve the problem of energy shortage. In this investigation, we design a kind of vaneless turbine originating from a Tesla turbine with a diameter and an air gap of 250 mm and 0.5 mm, respectively. Importantly, such a vaneless turbine removes the feature of an air outlet in the middle and adopts other ways of entering and leaving on both sides, so as to strengthen the rotor, because there is no need for a large hole in the middle of the rotor. Furthermore, the rotor dynamics characteristics of the vaneless turbine are calculated by six different modes. We also obtain the critical speed in different modes. Moreover, the flow field performances, such as the velocity and pressure of fluid (air), are investigated using the finite element simulation method. In addition, the bench test is built to obtain the output characteristics of a vaneless turbine. The maximum output torque is about 5.56 Nm at 992 rpm, and the maximal rotational speed of the vaneless turbine can reach 3200 rpm. Our work provides new ideas and guidance for the design and research of the new generation of the vaneless turbine.

## Introduction

Nowadays, in order to alleviate the energy shortage, energy conversion and energy storage engineering have been widely concerned. From nanoscale functional materials (Ren et al., 2022a; Zhang et al., 2022a; Ren et al., 2022b; Ren et al., 2022c; Ren et al., 2022d; Ren et al., 2022e; Ren et al., 2022f; Qin et al., 2022) to large engineering equipment (Zhang et al., 2022b; Lee and Hong, 2022; Li and Taghizadeh-Hesary, 2022), extensive energy conversion research studies have been developed. In recent years, compressed air energy storage has attracted significant focus (Fayziyev et al., 2022; Liang et al., 2022). Compressed air energy storage mainly uses the residual power compressed air at the low load of the power grid and stores it in the high-voltage sealed facilities to drive the gas turbine for power generation at the peak of power consumption. Compressed air energy storage technology is a new energy storage technology. Compared with other energy storage methods, compressed air energy storage has been considered a green and sustainable energy storage technology with unique characteristics of high capacity and long storage time, and its scale and cost are similar to those of a pumped storage power station (Wang et al., 2017). The efficiency of a complete charge–discharge cycle with several adiabatic compressed air energy storage configurations was analyzed by means of energy balance. The key factor to improve efficiency is to develop high-temperature heat storage (larger than 600°C) and high-temperature resistant materials for compressors (Hartmann et al., 2012). An average round trip energy efficiency (output electric energy/input electric energy) of 22.6% was achieved by Wang et al. (2016), and the maximum generator power of 430 kW was obtained during the discharge process. Moreover, in an energy storage system such as compressed air, the conventional turbine plays a key role, such as pneumatic motor, which converts the pressure energy of compressed air into rotating mechanical energy. However, the pneumatic motor needs regular maintenance and low energy efficiency (less than 65%). Therefore, there are still many restrictions on the use of pneumatic motors (Stoianovici et al., 2007; Tanaka et al., 2013; Deaconescu and Deaconescu, 2022).

Compared to pneumatic motors, the Tesla turbine has attracted abundant research because of its unique working principle (Song et al., 2017; Manfrida et al., 2018; Rusin et al., 2018). The Tesla turbine is a blade-free turbine driven by fluid viscous force, which was proposed by Nikola Tesla in 1913. The Tesla turbine is called a vaneless turbine because it applies the boundary layer effect instead of the traditional direct impact of fluid on turbine blades. In addition, Tesla turbines are also known as “boundary layer turbine,” “condensation-type turbine,” and “Prandtl layer turbine.” The principle of the Tesla turbine is the boundary layer effect of fluid. Influenced by viscous force, the fluid will form a thin boundary layer on the edge of a pipe wall or other objects. In the boundary layer, the velocity on the fixed surface is 0, and the farther away from the surface, the greater is the velocity. Using this effect, a group of disks can be driven by a high-speed liquid. Therefore, its efficiency is much higher than that of an ordinary blade turbine, and the theoretical efficiency can reach more than 85%. Recently, lots of investigations have been explored to study the characteristic of the Tesla turbine. For example, the flow field characteristics of the Tesla turbine were systematically studied by simulation calculations using three working fluids (Ciappi et al., 2019). Such a turbine possesses high output efficiency of about 69% at the rotational speed of 3000 rpm. A new procedure for the Tesla turbine using organic Rankine cycle applications was developed, which can estimate the expander size (Talluri et al., 2018). Furthermore, a system design method of the Tesla turbine was proposed to decide the decent parameters and geometric model of the Tesla turbine, which demonstrates that the Tesla turbine presents an excellent performance at low rotational speed (Ji et al., 2019). However, the gas circulation mode of the Tesla turbine determines the particularity of its structure explaining the tiny gap and middle opening as a gas outlet. Thus, the strength of the structure will be greatly reduced. Moreover, the modal is also the key parameter influencing the deformation during rotation, which is rarely investigated.

Therefore, in this work, a new vaneless turbine was designed. The simulation methods were conducted to study the structural characteristics, rotor dynamics properties, and internal gas flow field performances under different rotational speeds. Then, the output rotational speed and torque of such a vaneless turbine were monitored by the bench test.

## Simulation analysis

### The design of the vaneless turbine

**FIGURE 1:**
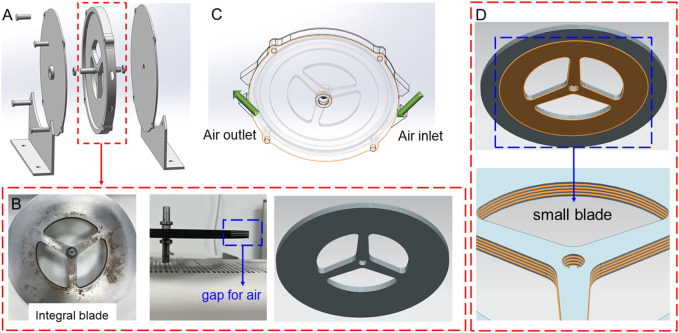
Demonstration of the designed vaneless turbine in this work: **(A)** exploded view of assembled structure, **(B)** structure of the assembled integral blade, **(C)** air flowing path, and **(D)** small blade in the assembled integral blade.

We proposed an extraordinary vaneless turbine originating from the Tesla turbine, and the overall assembly structure is shown in Figure 1A. The critical components are blade (demonstrated in Figure 1B), bearings, and shell, etc. Different from the traditional Tesla turbine, the inlet and outlet ports of the turbine are located on the edge of the disc rather than the middle outlet, as shown in Figure 1C. Thus, the middle of the blade is made solid, and then the weight of the blade is reduced through some holes. In particular, to retain a decent gap for the boundary layer effect between the air and blade, the gap is built by inserting a smaller blade with a thickness of 0.5 mm between each blade, which means the gap is 0.5 mm for the turbine, exhibited in Figure 1D. The diameters of the larger and middle blades are 250 mm and 200 mm, showing that the depth of the gap is 25 mm for the air. Furthermore, considering the mechanical properties and processing characteristics of the material, silicon steel was selected as a blade. For a better boundary layer effect, the blade is finally annealed and coated with insulating coating on both sides. The surface of the blade is smooth and clean without grease and rust. The coating is a mixture of inorganic components based on chromate, with a small amount of organic components added to improve the punching performance of the blade. The coating has a certain interlayer resistance. In a neutral atmosphere or weak reducing furnace atmosphere, the maximal stress relief annealing temperature, which is up to about 800°C, and the interlayer resistance will be reduced during annealing. The coating can withstand 300–550°C burning treatment. Finally, the assembled integral blade is obtained by gluing.

### Modal calculation of rotor dynamics

**FIGURE 2:**
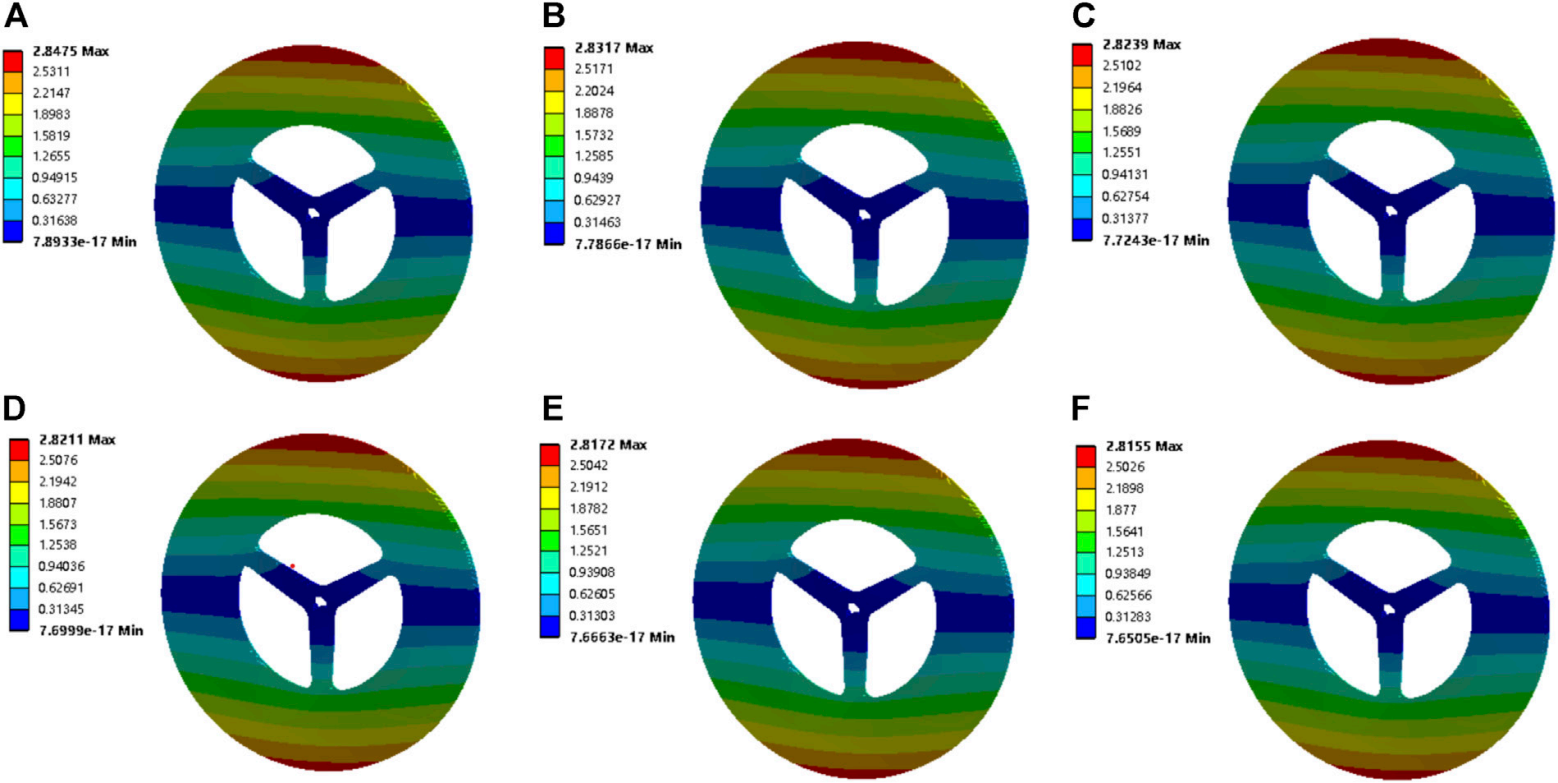
Deformation of the vaneless turbine by the first mode at **(A)** 500 rpm, **(B)** 1000 rpm, **(C)** 1500 rpm, **(D)** 2000 rpm, **(E)** 2500 rpm, and **(F)** 3000 rpm.

As a high-speed rotating part, the rotor dynamics characteristics of this vaneless turbine are crucial to evaluate its failure capability. Due to the manufacturing error, the center of mass of each micro-segment of the rotor generally deviates slightly from the rotation axis. When the rotor rotates, the centrifugal force caused by the aforementioned deviation will cause the rotor to generate lateral vibration. This vibration is extremely strong at some speeds, which are called critical speeds. In order to ensure that the machine will not resonate within the working speed range, the critical speed should be appropriately deviated from the working speed, for example, by more than 10%. The critical speed is related to the elasticity and mass distribution of the rotor. For a discrete rotating system with finite lumped masses, the number of critical speeds is equal to the number of lumped masses; for an elastic rotating system with a continuous mass distribution, there are infinitely many critical speeds. The most commonly used numerical method to calculate the critical speed of a large rotor support system is the transfer matrix method. The main points are as follows: first, the rotor is divided into several sections, and the relationship between the four section parameters (deflection, deflection angle, bending moment, and shear force) at the left and right ends of each section can be described by the transfer matrix of this section. In this way, the total transfer matrix between the cross-sectional parameters of the left- and right-end faces of the system can be obtained. Then, based on the boundary condition and the condition that there is a non-zero solution in the natural vibration, the critical speed of each order is obtained by the trial and error method, and then the corresponding vibration mode is obtained. Thus, the modes at different rotational speeds are calculated. A mode is the natural vibration characteristic of the structural system. The free vibration of the linear system is decoupled into *N* orthogonal single degree of freedom vibration systems, corresponding to *N* modes of the system. Each mode has a specific natural frequency, damping ratio, and mode shape. These modal parameters can be obtained by calculation or test analysis, and such a calculation or test analysis process is called modal analysis. Through the structural modal analysis method, the vibration characteristics of various modes of the mechanical structure in a certain vulnerable frequency range can be obtained, as well as the vibration response results of the mechanical structure in this frequency band and under the excitation of various internal or external vibration sources. Then, the modal parameters can be obtained by the modal analysis method and combined with relevant tests, and these unique parameters can be used for the structural redesign. We extracted the deformation under the first dielectric mode, as shown in Figure 2. One can find that the vaneless turbine presents a maximum deformation at 500 rpm. Importantly, the maximal deformation is about 2.8 mm, and the high rotational speed results in lower deformation which means the resonance amplitude will be small at high rotational speed for the first mode of the vaneless turbine.

**FIGURE 3:**
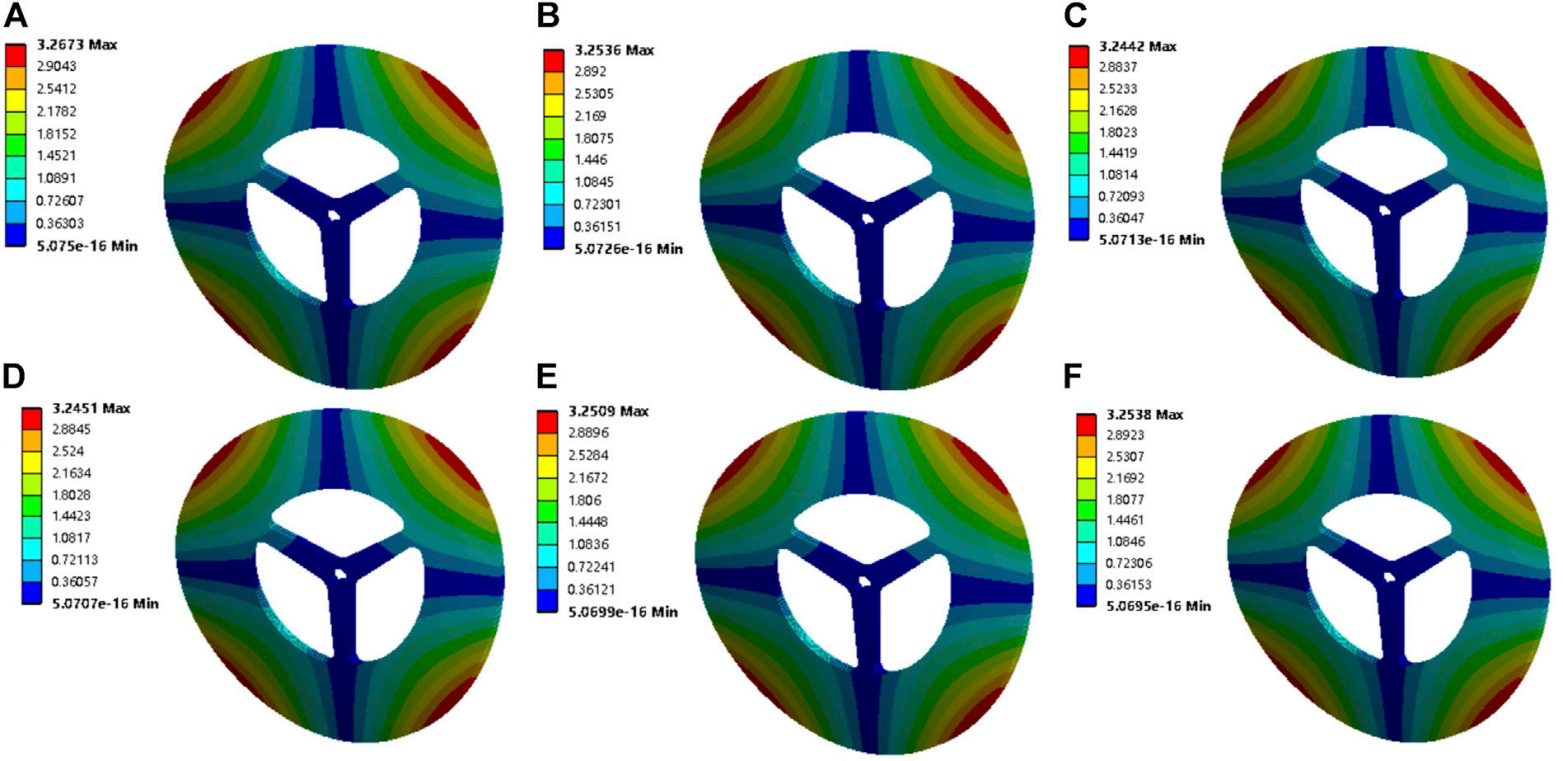
Deformation of the vaneless turbine by the sixth mode at **(A)** 500 rpm, **(B)** 1000 rpm, **(C)** 1500 rpm, **(D)** 2000 rpm, **(E)** 2500 rpm, and **(F)** 3000 rpm.

**FIGURE 4:**
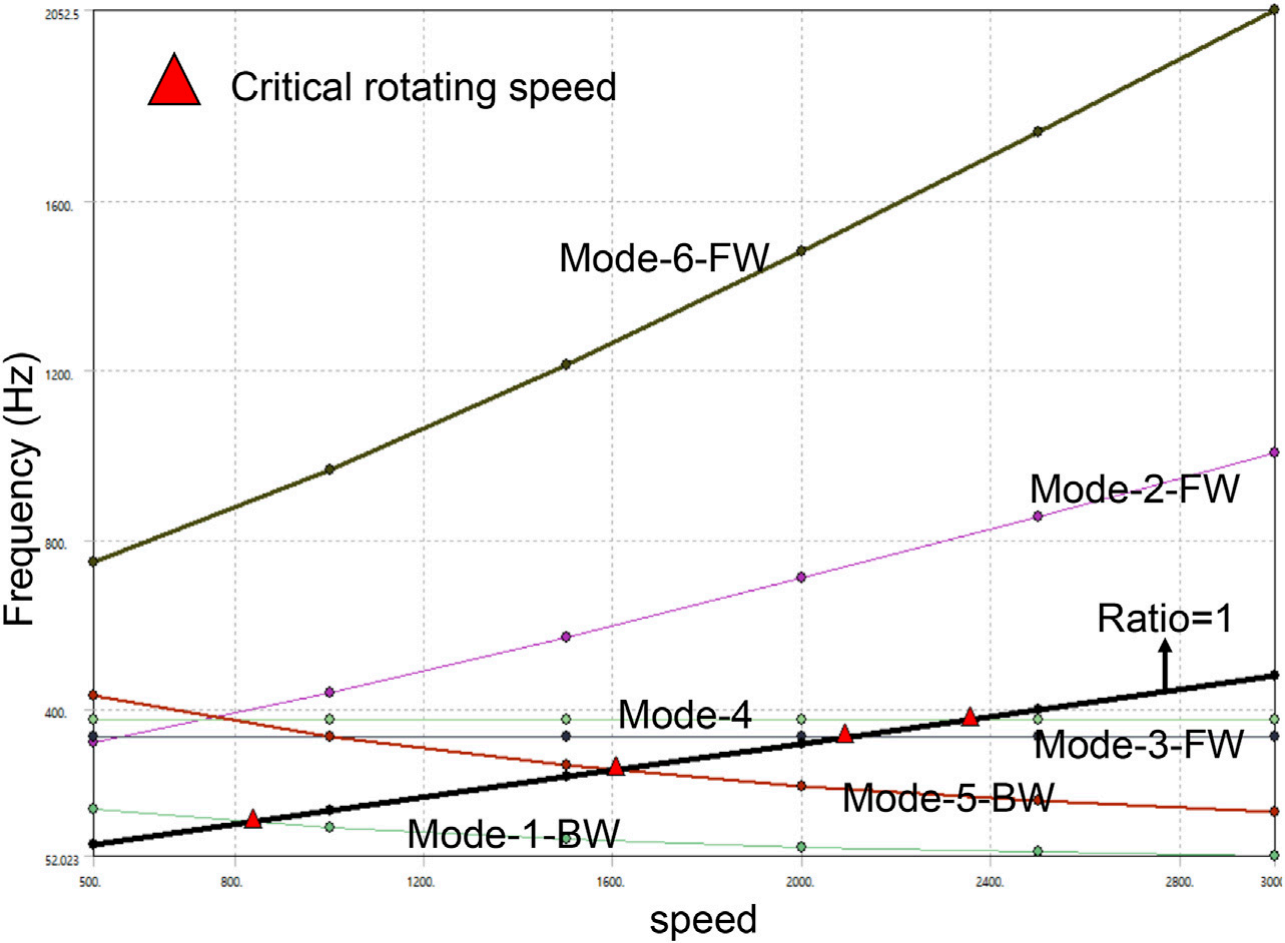
Calculated Campbell diagram of the vaneless turbine by the first six modes.

Then, the deformation under the sixth dielectric mode of the vaneless turbine at different rotational speeds is demonstrated in Figure 3. These induced deformations are obviously larger than those of the first modal. Moreover, the maximum deformation is still explored at 500 rpm. The maximal deformation is obtained about 3.2 mm for the vaneless turbine at these rotational speeds under the sixth mode. Differently, the high rotational speed suggests large deformation; thus, avoiding high-order frequencies at high rotational speeds is critical for the vaneless turbine. Moreover, the deformation of the vaneless turbine by second- to fifth-order mode is presented in Supplementary Figures S1, S4. Furthermore, the Campbell diagram of the vaneless turbine is exhibited in Figure 4. The FW and the BW are forward and backward rotations, respectively. In the figure, the horizontal axis is the rotational speed value, the vertical axis is the frequency value, and the slope of the slash of the zero crossing point is 1. The intersection of the slash and frequency line is the critical speed of the rotor on the abscissa. Evidently, as the rotational speed of the vaneless turbine increases, the natural frequency will also increase at second and sixth modes, while the third and fourth modes possess slight change, which means the torsional deformation occurs in modes 3 and 4, and the natural frequency remains basically stable. In addition, the rotational speed under the first and fifth orders is inversely proportional to the natural frequency.

In general, when the rotational speed of the rotating mechanism increases, the amplitude will increase greatly near some rotational speeds. This is because when the working speed occurs at the natural frequency, its transverse amplitude will increase greatly. The speed at this time is the critical speed. The critical speed of the first four modes is greater than the working speed, which indicates that the system has a sufficient vibration safety margin and will not resonate. However, when the working speed is greater than 2500 rpm, resonance may occur (at this time, the critical speed is less than the working speed). With the continuous increase in rotational speed, the natural frequency will not always increase, but also decrease, which may be due to the rotation softening effect and gyro effect.

### Flow simulation calculation

**FIGURE 5:**
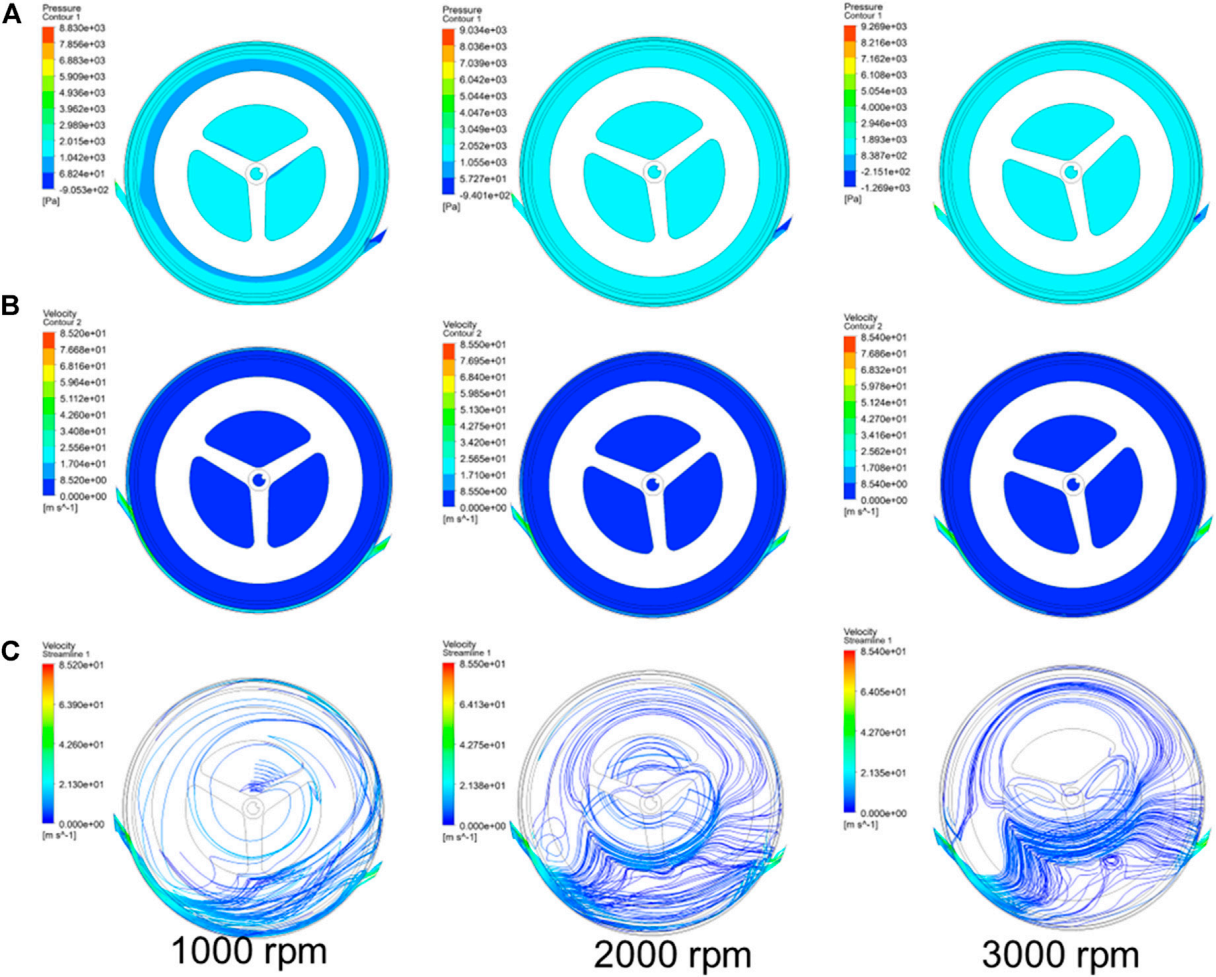
Obtained flow characteristics of the **(A)** pressure, **(B)** speed, and **(C)** streamline diagram of the vaneless turbine at different rotational speeds.

The flow characteristic is an important way to study the working performance of a turbine. Here, we calculated pressure, speed, and streamline diagram of the fluid (air) in the vaneless turbine by different rotational speeds. In Figure 5A, the pressure of the overall fluid in the vaneless turbine is demonstrated at 500 rpm–3,000 rpm. It can be seen that the maximum pressure has occurred at the lower rotational speed, which means the larger torque. In addition, the speed of the fluid at different rotational speeds is shown in Figure 5B, and the results are in accordance with the input parameters of speed. Moreover, the streamline diagram of the fluid in the vaneless turbine is also calculated in Figure 5C, and the results explain that the flow path of the fluid in the turbine does not return, thus showing a normal flow state.

## Experimental investigation

**FIGURE 6:**
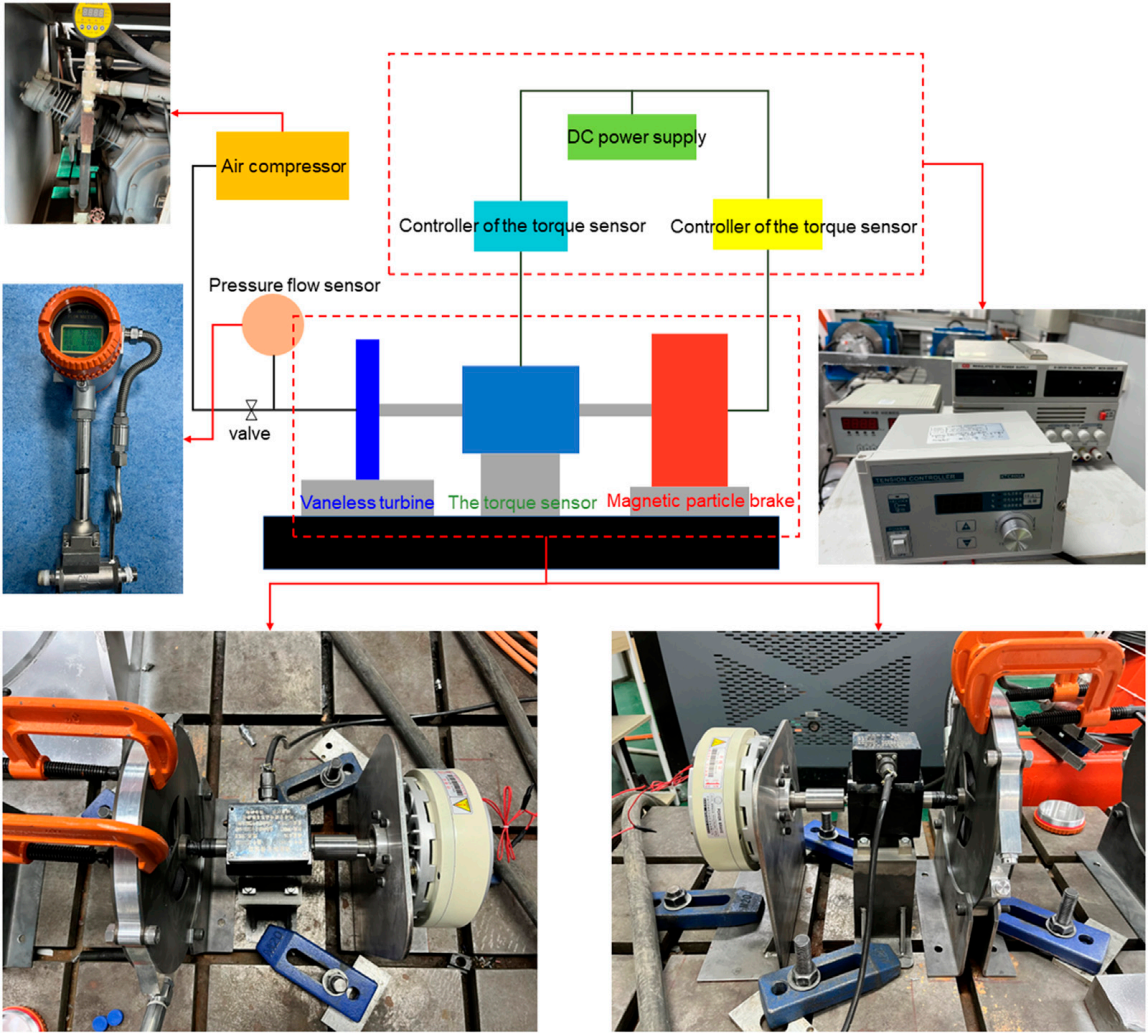
Test platform for output characteristics of the vaneless turbine.

In order to verify the actual output characteristics of such a vaneless turbine, we have built an experimental platform using the torque sensor, air compressor, and magnetic particle brake, etc., which are demonstrated in Figure 6. The air compressor is used as the boost for the turbine, which possesses a 3 MPa capacity. It is worth noting that the gas pipeline also can endure a maximum load of about 10 MPa. During the test, the inlet and outlet pressure and flow are monitored by the flow sensor, as shown in Figure 1. The shafts of the turbine, torque sensor, and magnetic particle brake are installed at the same height, and their shafts are connected as a shaft with a sleeve to rotate. After the turbine is driven, the magnetic particle brake acts as a towed motor feedbacking the torque from the turbine. The braking force of the magnetic particle brake is regulated by the current. The middle torque sensor can read the output speed and torque of the turbine.

**FIGURE 7:**
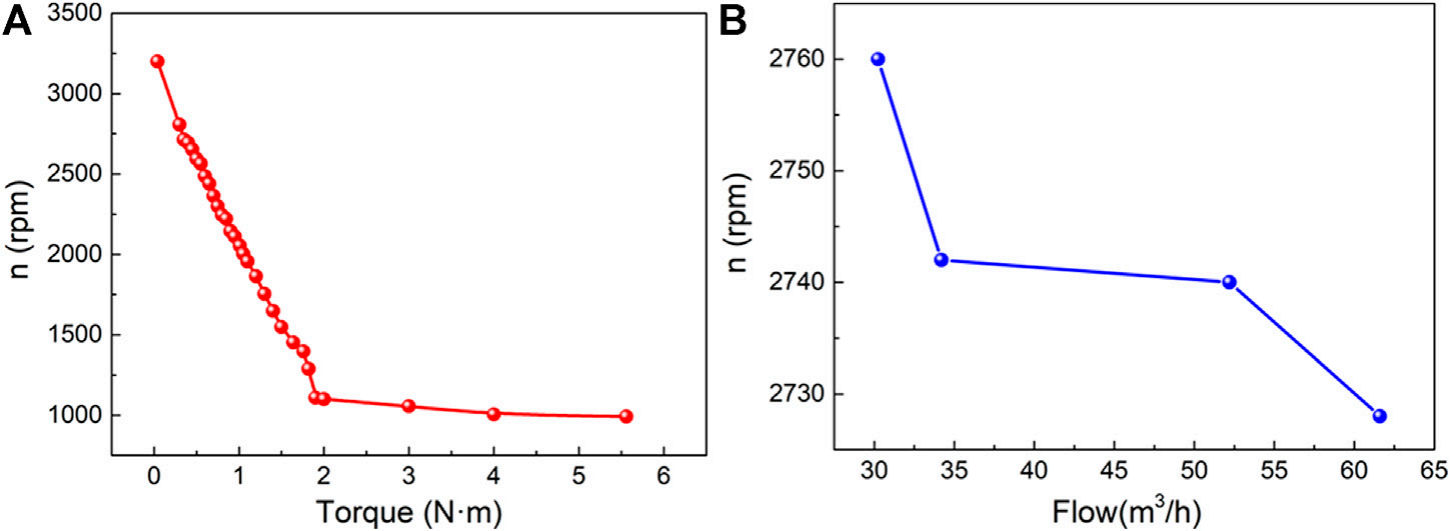
**(A)** Output of the rotational speed by different torques of the turbine; **(B)** output of the speed by different flows in the turbine.

Under the no-load condition, with the output of compressed gas from the air compressor, the vaneless turbine takes about 150 s from the start to steady state. Also, the obtained maximum rotational speed is up to 3200 rpm. Then, the current of the magnetic particle brake is increased, and the output rotational speed decreases, as shown in Figure 7A. One can find that the rotational speed drops rapidly within 2 Nm, while the maximum output torque is about 5.56 Nm at 992 rpm. The response of the output rotational speed to the input flow is also investigated, as shown in Figure 7B, demonstrating that the increase of flow can reduce the output rotational speed because of the decreased rotational speed of input gas.

## Conclusion

The gas in the Tesla turbine usually enters from the side and exits from the middle, but this structure makes the turbine blades easy to be deformed under high pressure. Based on this, our investigation proposes a vaneless turbine with an inlet and outlet on both sides, so that the strength of the blades can be greatly improved. Then, in order to study its resonance characteristics, the eigenfrequencies at different speeds and the critical speeds at different modes are calculated. The fluid characteristics at different speeds are also monitored by finite element analysis. Furthermore, the turbine sample is made, and the bench test is also built to explore the output characteristic of such a vaneless turbine. This vaneless turbine presents a novel torque of about 5.56 Nm at 992 rpm and a no-load rotational speed of about 3200 rpm.

## Data Availability

The raw data supporting the conclusion of this article will be made available by the authors, without undue reservation.
