# Supplementary material for: A new type of bladeless turbine for compressed gas energy storage system
Source: Front Chem. 2022 Sep 26;10:1013473. doi: 10.3389/fchem.2022.1013473 (PMC9548558; doi:10.3389/fchem.2022.1013473)
Supplement: Supplementary file 1 [file DataSheet1.docx]

**A New Type of Bladeless Turbine for Compressed Gas Energy Storage System**

Qin Wang^1^, Zhengyang Zhu^2,*^ Wei Chen^3^ and Yang Zhou^3^

^1^Key Laboratory of Energy Thermal Conversion and Control of Ministry of Education, School of Energy and Environment, Southeast University, Nanjing 210096, China

^2^School of Mechanical Engineering, Wanjiang University of Technology, Maanshan 243031, China

^3^Jiangsu Jiaoke Energy Technology Development Co., Ltd, Nanjing 210017, China

E-mail: Zhengyang_Zhu@126.com


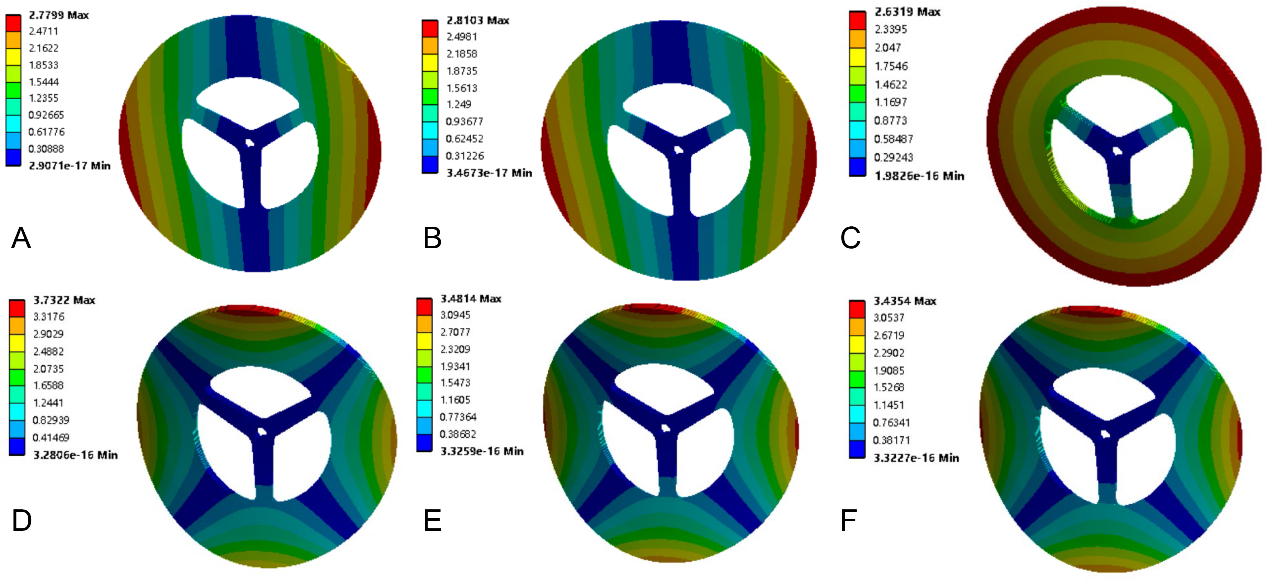


**FIGURE S1.** The deformation of the vaneless turbine by the second mode at (A) 500 rpm, (B) 1000 rpm, (C) 1500 rpm, (D) 2000 rpm, (E) 2500 rpm and (F) 3000 rpm.


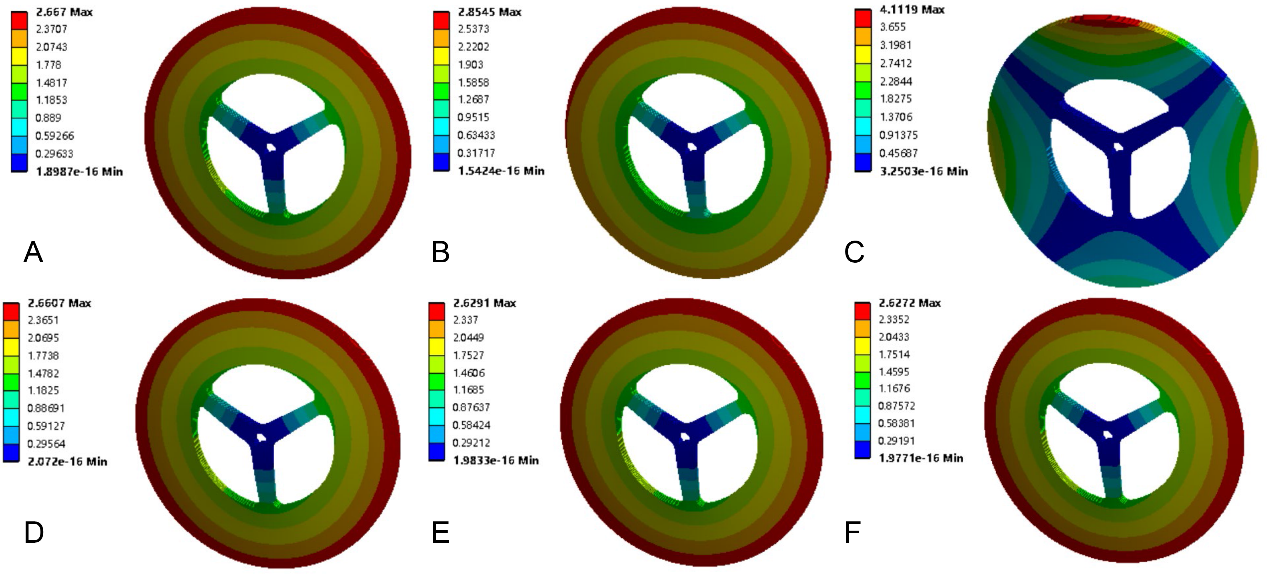


**FIGURE S2.** The deformation of the vaneless turbine by the third mode at (A) 500 rpm, (B) 1000 rpm, (C) 1500 rpm, (D) 2000 rpm, (E) 2500 rpm and (F) 3000 rpm.


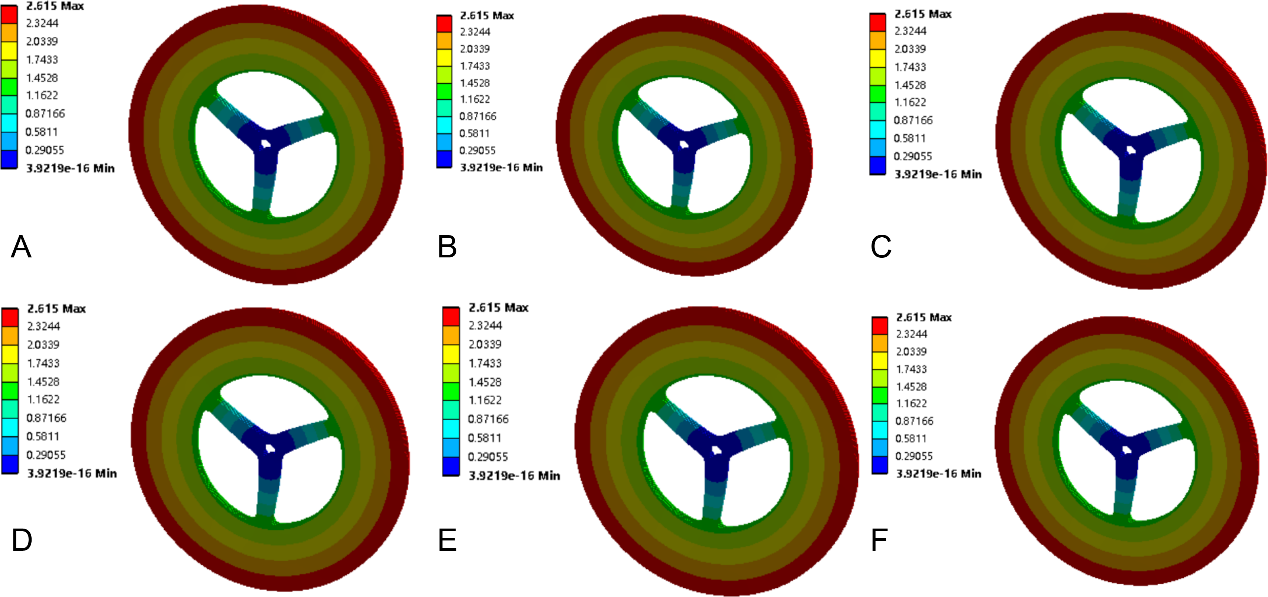


**FIGURE S3.** The deformation of the vaneless turbine by the fourth mode at (A) 500 rpm, (B) 1000 rpm, (C) 1500 rpm, (D) 2000 rpm, (E) 2500 rpm and (F) 3000 rpm.


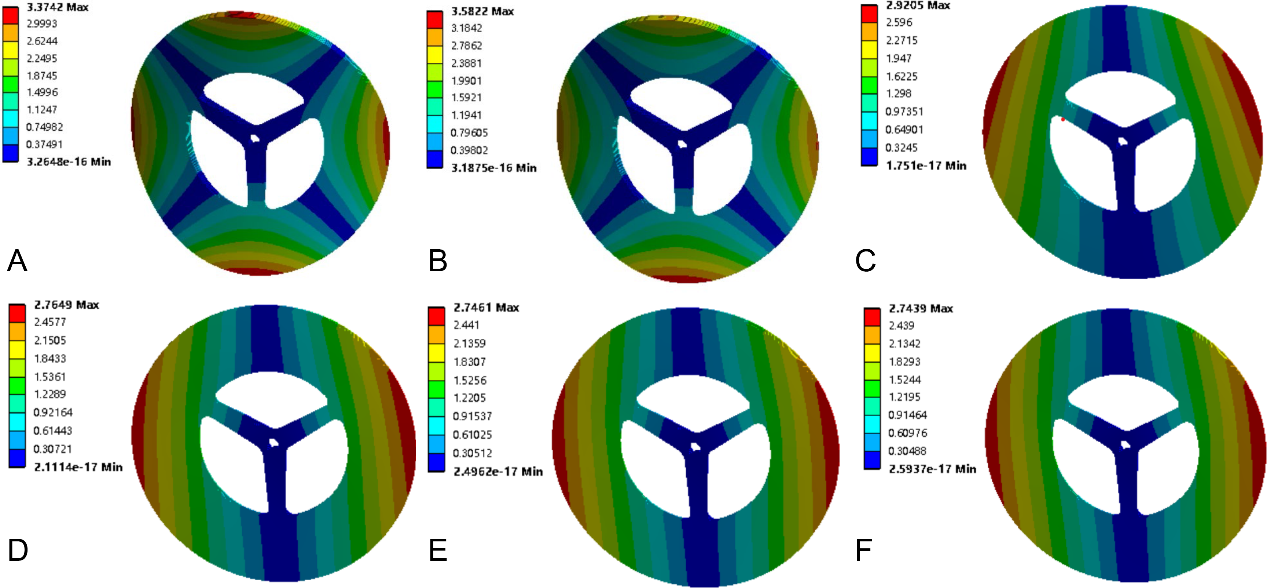


**FIGURE S4.** The deformation of the vaneless turbine by the fifth mode at (A) 500 rpm, (B) 1000 rpm, (C) 1500 rpm, (D) 2000 rpm, (E) 2500 rpm and (F) 3000 rpm.
